# Removal of an Atypical Foreign Body Using Flexible Bronchoscopy Under Local Anesthesia

**DOI:** 10.7759/cureus.56938

**Published:** 2024-03-26

**Authors:** Nahid Zaghba, Fatima Ezzahra Haouassia, Khadija Chaanoune, Hanane Benjelloun, Najiba Yassine

**Affiliations:** 1 Pulmonology Department, Ibn Rochd University Hospital, Casablanca, MAR

**Keywords:** denture base resin, soft bronchoscopy, extraction, foreign object, local anesthetic

## Abstract

While uncommon among adults, the act of inhaling a foreign object is a grave incident that might potentially endanger one's life or result in substantial repercussions. A 43-year-old patient with a history of asthma and epilepsy from infancy appeared with worsening respiratory distress and the presence of purulent secretions one week following an epileptic seizure.

The chest X-rays and abdominal ultrasound revealed no anomalies. A bronchoscopy performed with local anesthesia enabled clear vision of the foreign object, and its removal was successfully executed, eliminating the need for a more invasive procedure. Bronchoscopy is crucial for both diagnosis and treatment, particularly in cases where there is a suspicion of inhalation of a foreign object that cannot be seen on X-rays. However, X-rays can still be useful for detecting radiopaque foreign objects or for identifying indirect symptoms of their existence.

## Introduction

Foreign body inhalation in adults is a rare yet potentially life-threatening event that requires prompt recognition and appropriate management strategies [1,2]. While more commonly associated with pediatric populations, foreign body inhalation remains a significant concern in adults, with diverse presentations and associated risks [3-5]. Given its effectiveness and minimally invasive nature, bronchial endoscopy has emerged as the preferred diagnostic and therapeutic approach for foreign body removal in adults. Surgical intervention may be necessary in cases where bronchoscopic extraction fails or is contraindicated due to anatomical considerations or patient factors [3].

Our case underscores the successful extraction of dentures via flexible bronchoscopy under local anesthesia, exemplifying the utility of minimally invasive techniques in managing foreign body inhalation in adults. By highlighting this approach, we contribute to the evolving literature aimed at refining clinical practices and optimizing patient outcomes in this challenging clinical scenario.

## Case presentation

**Figure 1:**
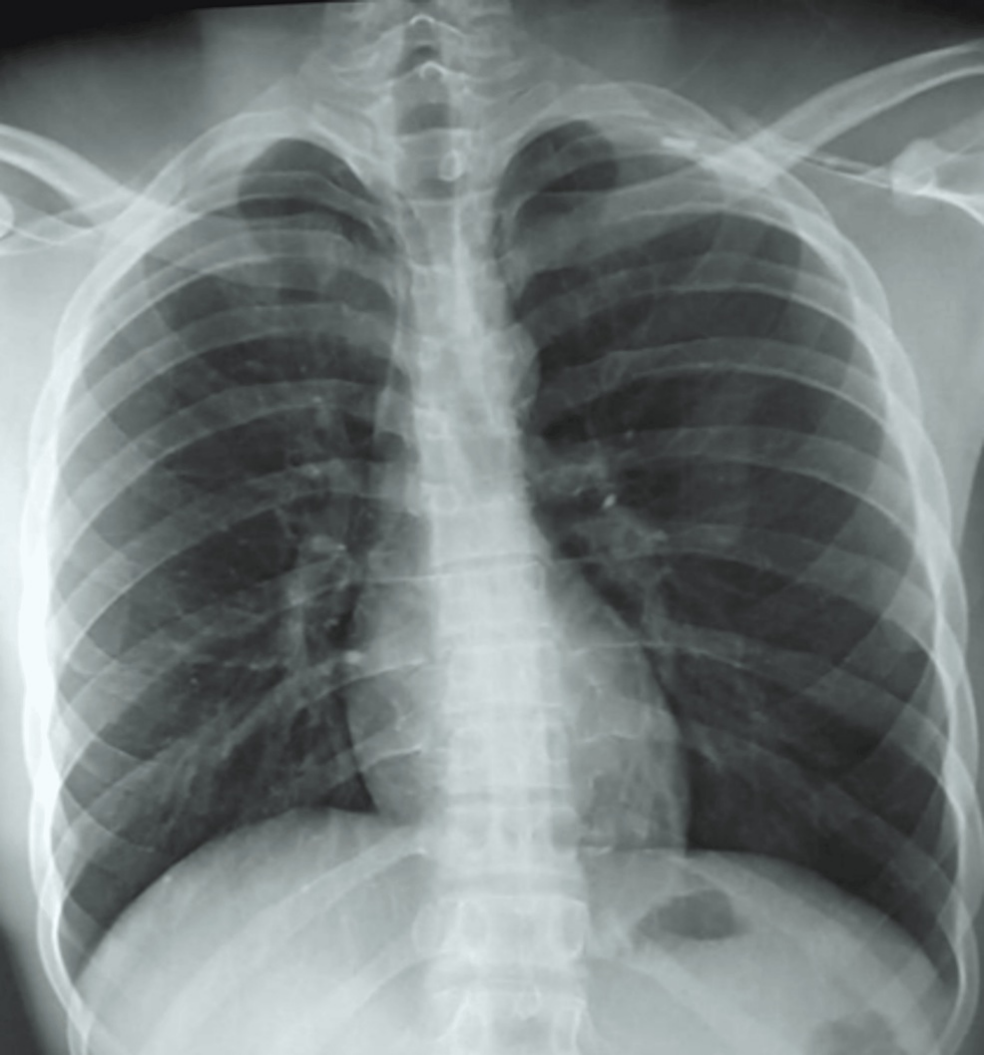
X-ray Thorax The image shows normal thoracic radiography outside of a thoracic distension.

The patient, aged 43 years, has been under surveillance for epilepsy since childhood and is currently undergoing treatment for epilepsy. In addition, he had a history of respiratory wheezing issues. Despite adherence to prescribed medication, the patient experienced a tonic-clonic crisis with urine discharge and an epileptic attack while outdoors. Post-seizure, the patient expressed concern about tooth loss during the event (after waking up from his epileptic seizure, he couldn't find his teeth), despite the absence of concurrent respiratory symptoms. Prompted by worsening respiratory symptoms and the presence of purulent secretions a week later, the patient underwent a chest X-ray (Figure 1), which revealed thoracic distension but no discernible abnormalities.

Subsequent spirometry demonstrated a severe obstructive ventilatory disorder reversible under beta-2 medications, prompting optimization of treatment. However, persistent symptoms led the patient to seek consultation at the University Hospital Center Ibn Rochd in Casablanca.

**Figure 2:**
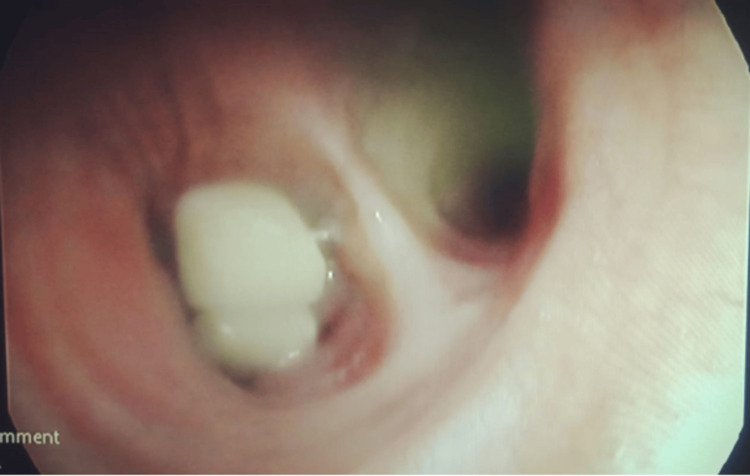
Bronchoscopy image A flexible bronchoscopy reveals the resin teeth at the right main bronchus entry.

**Figure 3:**
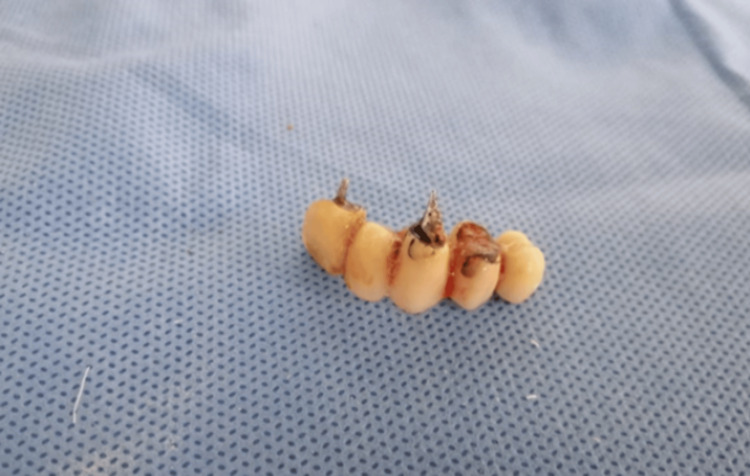
Dentures made from resin recovered after the procedure

At the hospital, a comprehensive interrogation and physical examination were conducted, revealing no anomalies, notably the absence of foreign body aspiration. Further examination ruled out additional risk factors for inhalation, including disorders of regurgitation, neuromuscular pathology, or alcoholism. The patient exhibited stable hemodynamics and respiratory function upon clinical assessment. Subsequently, a flexible bronchoscopy under local anesthesia was performed, revealing the presence of a foreign object at the right main bronchus entry (Figure 2), which was successfully removed without resorting to more invasive procedures (Figure 3). The procedure lasted 20 minutes.

Post-removal radiological imaging showed normal findings, with immediate favorable evolution noted, particularly following temporary treatment with antibiotics and corticosteroids. It is pertinent to mention that the resin, in which the patient believed his teeth were lost, lacks radioopacity, necessitating endoscopic visualization for diagnosis rather than relying solely on radiography.

## Discussion

Accidental inhalation of a foreign object is an ordinary occurrence among children, with a male predominance [1]. In adults, intra-bronchial foreign bodies are rare, often difficult to diagnose, and constitute a diagnostic and therapeutic emergency [2]. Foreign objects frequently encountered in cuisine include peanuts, peanut seeds, and sunflower seeds. Occasionally, the items in question may consist of coins or metallic objects [3,4]. The characteristics of intra-bronchial foreign bodies differ based on geographical regions, dietary patterns, and attire [1,5,6]. Foreign objects frequently encountered in cuisine include peanuts, peanut seeds, and sunflower seeds. Occasionally, the items in question may consist of coins or metallic objects [3,4]. The characteristics of intra-bronchial foreign bodies differ based on geographical regions, dietary patterns, and apparel (clothing) [1,5,6].

The risk of ingesting foreign items grows in children when they can turn their palms downward (pronation) and are inclined to put objects in their mouths. This hazard is exacerbated during meals due to inefficient mastication and the absence of coordination between swallowing and closing the glottis [7]. During adulthood, a decline in attentiveness, intense emotions while eating, and working in specific professions (such as scouts, rope sellers, and carpenters) are frequent causes of inhaling foreign objects into the bronchial tubes. Additionally, elderly individuals are more prone to inhaling foreign bodies while sleeping due to inadequate toothbrushing, a weakened cough reflex, and difficulty swallowing [1,7].

The presence of a foreign body aspiration, which could impact the prognosis, is a crucial component of the diagnostic process. This syndrome is characterized by access to suffocation with coughing and occasionally cyanosis, tugging, or cornage. Diagnosis may be delayed for years, though, as this illness may go unnoticed or untreated [2,7]. The lack of attention to an object trapped within the bronchial tubes leads to its entrapment, resulting in frequent and distressing respiratory symptoms. The persistent nature of inflammatory processes results in the development of a protective layer that conceals the foreign object, making it difficult to detect during endoscopy [1,2].

When a foreign body is in the bronchi, the most common side effects are pleurisy, dyspneic bronchopneumonia, pulmonary abscesses, pneumothorax, and obstructive emphysema, which is often linked to recurrent pneumopathy in the same area [8]. The pneumomediastinum is exceptional [9]. Complications may arise if the period between inhaling the foreign object and removing it is extended beyond seven days. Besides mechanical issues, the presence of a foreign object can also lead to the development of a granulomatous reaction through the production of new connective tissue. This reaction can lead to the degradation of cartilage, the expansion of the bronchial tubes, the formation of fibrous tissue, or even excessive infection in the blocked area [10].

Detecting non-dental foreign objects in X-rays can be challenging [2], which highlights the crucial role of flexible endoscopy in the diagnostic procedure [11]. The identification of the alien body was not possible in our situation due to its radio-transparent nature and cannot be commented on due to the lack of computed tomography. A diagnostic bronchial fibroscopy is necessary in cases where there is uncertainty about the presence of a foreign body in the bronchial tree, even if the patient's symptoms and imaging results appear normal. The purpose of this is to prevent the development of parenchymal lesions [2].

In terms of therapeutic care, this observation highlights the need for flexible bronchoscopy, which is frequent enough to remove most foreign bodies, even when asphyxia forms are an urgent indication of de-obstruction and rigid bronchoscopy techniques. In fact, flexible endoscopy is becoming more common for the removal of foreign things when there are no symptoms of respiratory distress. These items can be gathered with a straightforward pinch or a special trap. The flexible bronchoscope offers more accessibility and cost-effectiveness compared to rigid endoscopy. However, the successful extraction of foreign bodies with this method relies heavily on the surgeon's expertise and proficiency [12,13]. Currently, there is no agreed-upon treatment approach for removing a trachea-bronchial foreign body, and the teams' experience plays a major role in this decision. The preferred method is still rigid bronchoscopy, performed under general anesthesia [7,14]. Nevertheless, there are certain drawbacks, including the requirement for general anesthesia and the possibility of increased morbidity in older patients.

If endoscopic extraction is unsuccessful, surgeons may recommend aggressive or conservative surgery as a final resort [2,7]. In this instance, the flexible bronchoscopy extraction under local anesthesia was accomplished without incident, avoiding the patient receiving a surgical operation.

## Conclusions

When foreign objects are inhaled into the bronchial tubes, they can cause permanent damage to the lung tissue. The most effective way to deal with this is to prevent it from happening in the first place. Immediate bronchial endoscopy to detect an intrabronchial foreign object is necessary for both diagnostic and therapeutic purposes when the mere notion of a penetration syndrome (foreign body aspiration) occurs and to stop any problems that could happen if there is one. The patient's persistent purulent secretion prompted our recommendation for flexible bronchoscopy. We advise performing this technique with the utmost caution and under local anesthesia alone, which effectively eliminates the need for surgery.
